# Unique Characteristics of Pulse-Echo Sensing Systems for Ultrasonic Immersion Testing in Harsh Environments

**DOI:** 10.3390/s24237748

**Published:** 2024-12-04

**Authors:** Gaofeng Sha, Andrew R. Bozek, Bernhard R. Tittmann, Cliff J. Lissenden

**Affiliations:** Department of Engineering Science and Mechanics, Penn State, University Park, PA 16802, USA; gaofeng.sha@cptc.edu (G.S.); arb6058@psu.edu (A.R.B.); brt4@psu.edu (B.R.T.)

**Keywords:** nondestructive testing, ultrasonics, harsh environment, aluminum nitride, finite element modeling

## Abstract

Ultrasound is an excellent way to acquire data that reveal useful information about systems operating in harsh environments, which may include elevated temperature, ionizing radiation, and aggressive chemicals. The effects of harsh environments on piezoelectric materials have been studied in much more depth than the other aspects of ultrasonic transducers used in pulse-echo mode. Therefore, finite element simulations and laboratory experiments are used to demonstrate the unique characteristics of pulse-echo immersion testing. Using an aluminum nitride piezoelectric element mounted on a vessel wall, characteristics associated with electrode thickness, couplant, backing material, and an acoustic matching layer are investigated. Considering a wave path through a vessel wall and into a fluid containing a target, when the travel distance in the fluid is relatively short, it can be difficult to discern the target echo from the reverberations in the vessel wall. When an acoustic matching layer between the vessel wall and the fluid does not suffice, a simple subtractive signal-processing method can minimize the reverberations, leaving just the target echoes of interest. Simulations and experiments demonstrate that sufficient target echoes are detected to determine the time of flight. Furthermore, a simple disc-like surface anomaly on the target is detectable.

## 1. Introduction

Ultrasonic nondestructive testing (NDT) of components and structures that operate in harsh environments is a challenging endeavor that adds to the safety and reliability of the operating systems, whether for power generation, propulsion, chemical processing, manufacturing, or other purposes. Harsh environments could include extreme temperatures, harmful chemical reactions, and radiation. We will consider an environment to be harsh if it requires special consideration of the materials comprising the ultrasonic sensing system. Specifically, the primary considerations are as follows: (i) active piezoelectric (of course there are other types of active transducer materials, but, in this work, we limit consideration to piezoelectrics) material selection, (ii) how the transducer design and wave path affect the received signals, and (iii) how the received ultrasonic signals can ultimately be used to provide information about the system operations. While the first of these, material selection, and how the transducer materials tolerate elevated temperatures and irradiation have been studied extensively [1,2,3,4,5], the second consideration regarding how the transducer design and wave path affect the received signals is explored in detail in the present work.

**Figure 1:**
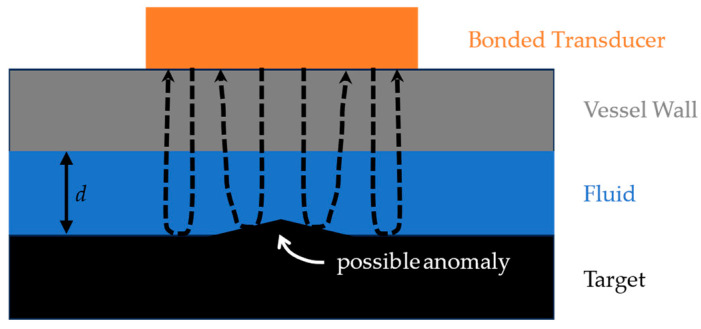
Pulse-echo ultrasound immersion test configuration.

The two-fold problem addressed herein is to determine the distance, *d*, traveled through a fluid to a target and to detect anomalies on the target’s surface. The transducer is mounted on the exterior of a vessel wall; therefore, the wave path is through the vessel wall and into the fluid. The waves reflect off the target and travel back through the fluid and the vessel wall, eventually being detected by the transducer in pulse-echo mode. A simplified schematic of the wave path is depicted in Figure 1. An NDT or process monitoring methodology could be based on the time of arrival (TOA) of the target’s echo being dependent upon the distance *d* and/or that anomalies may cause scattering or additional echoes. The pulse-echo sensing system could be adopted for a variety of purposes. In fact, the target can be any media having a sufficiently large acoustic impedance mismatch with the fluid to create a reasonable reflection. It could be a metal submerged in the liquid, the far end of a vessel/container, or a liquid–gas boundary within the vessel. Next, we discuss some applications for these types of pulse-echo ultrasound measurements.

Liquid metal fast reactors based on sodium or lead are being considered for the next generation of commercial nuclear power. However, the liquid metal is optically opaque, making the visual inspection of reactor components difficult. Under sodium viewing was investigated in [6,7]. Pulse-echo measurements from an ultrasonic transducer located below the tank bottom could determine the height of the liquid metal coolant, or a transducer on a tank side wall could locate mechanical components submerged in the liquid metal. Another application for this transducer design is for pipes or tanks in steam plants. Likewise, the pulse-echo transducer design can be applied to detect corrosion, scale deposits, or other blockages on either side of the inner radius of a pipe, while requiring access to just one side of the pipe.

In order to cover a broad range of harsh environments, we investigate aluminum nitride (AlN) as the active piezoelectric material for the transducer. Single crystal AlN does not have a phase transformation until its melting temperature at above 2000 °C. The elastic and electromechanical properties also remain stable at high temperatures [8]. Studies have shown that AlN can operate at temperatures of 1000 °C and gamma radiation doses of 18.7 MGy [2], see also [3].

Our research objective is to assess the characteristics of a pulse-echo ultrasonic test setup to remotely and nondestructively detect anomalies on the surface of an immersed target. A significant underlying task is to locate the position of the target’s surface. The model pulse-echo problem is described in Section 2, where finite element modeling of the transducer is employed to understand how its operation will affect the pulse-echo measurements. The laboratory setup is also described in Section 2. Since our interest is in the characteristics of a test setup appropriate for harsh environments, rather than materials selection, ambient laboratory conditions suffice and are used for convenience. The results in Section 3 are A-scans (wave amplitude in volts as a function of time at the transducer position). Both finite element model results and experimental results are presented for balance. We analyze the effect of backing material, electrode thickness, the solid couplant layer, and grain coarsening. In addition, we analyze the effect of a matching layer and signal processing to improve the clarity of the target echo. Finally, we demonstrate the detection of a surface anomaly on the target.

## 2. Materials and Methods

In this section, we first describe the pulse-echo ultrasound system alluded to in Figure 1 and then use finite element modeling to demonstrate how some of its features that are ancillary to the active piezoelectric element affect its performance. Specifically, with regard to the transducer, the effect of backing material, the electrode thickness, and the coupling media are illustrated. A description of the laboratory setup for pulse-echo ultrasonic immersion testing brings this section to a close.

### 2.1. Pulse-Echo Ultrasound System

**Figure 2:**
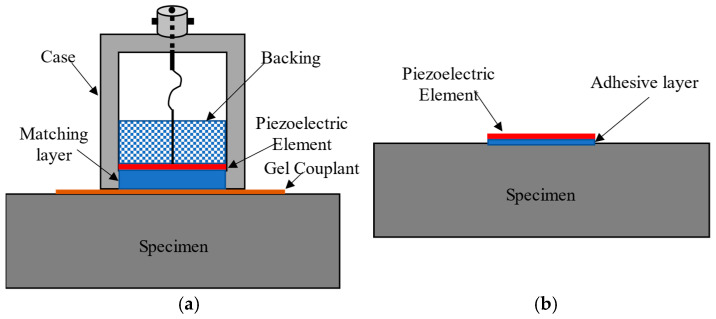
Typical ultrasonic transducers: (**a**) gel-coupled contact transducer and (**b**) bonded wafer.

High-frequency ultrasonic contact transducers typically consist of an active element, backing material, and a matching layer protected by a case, as shown in Figure 2a. Material selection for the components of transducers that operate in harsh environments is a critical first step. Previous studies demonstrate the capabilities of AlN transducers to operate in the MIT research reactor [2]. Figure 2b shows an alternative to the contact transducer popularized as stay-in-place sensors [9] for structural health monitoring applications. This study will evaluate stay-in-place AlN transducers having a piezoelectric element coupled directly to the vessel wall.

The harsh environment plays a major role in selecting the couplant because adhesives generally have poor tolerance for elevated temperatures and radiation. Moreover, liquid or gel couplants are not viewed as viable solutions for stay-in-place transducers. Thus, we investigate the extreme cases of phenyl salicylate and braze as the couplant between the transducer and the vessel wall. We recognize spray deposition [10] as an alternative method to avoid the need for a couplant but do not explore it here due to our choice of AlN as the active element. In loosely related work, Baba et al. [1] tested adhesively bonded lithium niobate wafers up to 1100 °C.

The laboratory tests are preliminary In nature and are not conducted in a harsh environment. Finite element models are run to support the laboratory testing. In these room-temperature tests, the fluid is simply water, and the target is a flat metal plate.

### 2.2. Modeling the Ultrasound System

Multiphysics modeling of ultrasonic testing based on piezoelectric transducers has been reported in the literature [11,12,13], and one of the commercial software packages used is COMSOL Multiphysics^®^ (Comsol.com). In this study, the whole system, including the transducer, driving circuit, and the test articles, is modeled to mimic the pulse-echo measurement in the experiment. This requires multiple physical field couplings and, thus, multiple interfaces are used in COMSOL. For example, the Piezoelectricity interface is used for ultrasonic transducers, while the Solid Mechanics interface is employed for the vessel wall and target (see Table 1).

Time domain analysis is used to model the pulse-echo signal in the approximate time range of 0–14 μs. The through-thickness resonant frequency of the AlN transducer is nominally 10 MHz, and for each component in the solid mechanics domain, there are 10 elements per wavelength (λ = *c*_L_/*f*, where *c*_L_ is the longitudinal wave speed and *f* is frequency). Incoherent measurement system noise has not been included in the simulation, although this could be added as random noise injected into both the input and output signals.

#### 2.2.1. Reduction to 2D Model

The AlN wafer has a square footprint, which requires a 3D model to accurately represent the geometry and a long computation time to simulate wave propagation. Therefore, we use an approximate 2D model by assuming an axisymmetric wavefield to reduce computation time. A comparison study confirms that this 3D model can be simplified into a 2D axisymmetric model approximation (called 2D model below).

**Figure 3:**
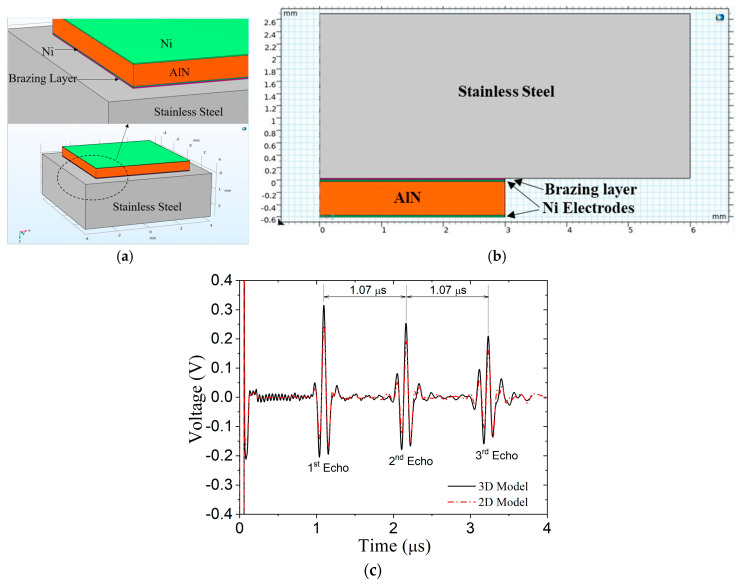
Finite element models: (**a**) 3D, (**b**) 2D axisymmetric, and (**c**) comparison of received signals from 3D and 2D axisymmetric models.

The geometries of the transducer and the vessel wall for the 3D model and 2D model are listed in Table 2, and corresponding material properties are provided in Table 3. In the 3D model, the transducer is brazed to the vessel wall, as shown in Figure 3a, and wave propagation is in the z-direction (vertical). The 2D model shown in Figure 3b is inverted. The left edge of the model is the assumed axis of rotational symmetry, where r = 0.

The transducer is driven by a negative electrical square pulse of 100 V with 50 ns duration that resembles one of our ultrasound instruments (OmniScan). This same driving signal is applied to all models unless otherwise specified. The generated ultrasonic wave propagates from the transducer through the vessel wall and is reflected back to the transducer. The received signals (known as an A-scans) for the 3D and 2D models are compared in Figure 3c. The signals illustrate that multiple reflections occur as the wave bounces back and forth in the wall/transducer model. No internal damping is used for the wall, and there is no viscous backing material, so the reverberations in the wall are substantial. It is evident that the 2D model faithfully reproduces the 3D model results, with the exception that the peaks are somewhat truncated. The difference in amplitude is most likely due to the difference in the transducer surface area; it is 36 mm^2^ in the 3D model and just 28 mm^2^ in the 2D model. This difference could be accounted for by increasing the voltage applied to the transducer in the 2D model, but we chose not to. The 2D model enables us to simulate the entire wave path and consider variations in the system and, therefore, will be used in the remainder of the simulations.

#### 2.2.2. Effect of Backing

**Figure 4:**
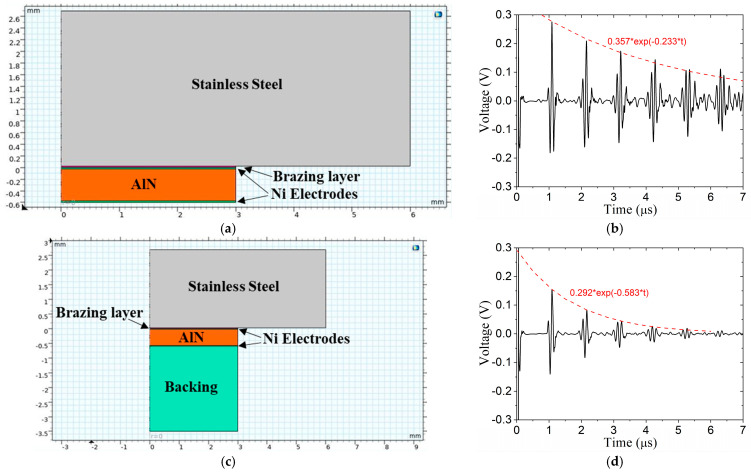
Backing material affects signals: (**a**) model with no backing, (**b**) A-scan for transducer with no backing, and (**c**) model with backing, (**d**) A-scan for transducer with backing. Properties of backing material given in Table 3.

The first question we ask the model to decipher is as follows: to what extent does backing material dampen the reverberations in the vessel wall? The backing material selected is a cured silicon/tungsten (Si-W) putty mixture. The backing layer is five-times as thick as the AlN transducer. Laboratory experiments were conducted to estimate the material properties provided in Table 3. The 2D models and received A-scan signals are shown in Figure 4 for a transducer with no backing (Figure 4a,b) and a transducer with backing (Figure 4c,d). The backing material is quite effective at attenuating the reverberations; in fact, the amplitude of the sixth echo is almost in the noise with the backing but still substantial with no backing. In addition, the backing affects the shape of the wave packet. Without backing, the positive peaks are larger than the negative peaks, while with backing, the positive and negative peaks are similar. Moreover, the backing material absorbs a significant amount of the energy in the first wave packet. Exponential equations were regressed to the reverberations in Figure 4b,d. The backing material increases the attenuation coefficient from 0.233 to 0.583 (a factor of 2.5). The large acoustic impedance mismatch between the wall and the surrounding air tends to keep the ultrasonic energy in the wall; however, when there is liquid in the vessel, more energy will be transmitted into the liquid. Despite the effectiveness of the backing material, upcoming simulations will not use a backing material because it reduces the initial wave packet and it does not dampen out the reverberations soon enough, as will become apparent in the results. On the other hand, there are many applications where a backing material will provide high value.

#### 2.2.3. Effect of Electrode Thickness

**Figure 5:**
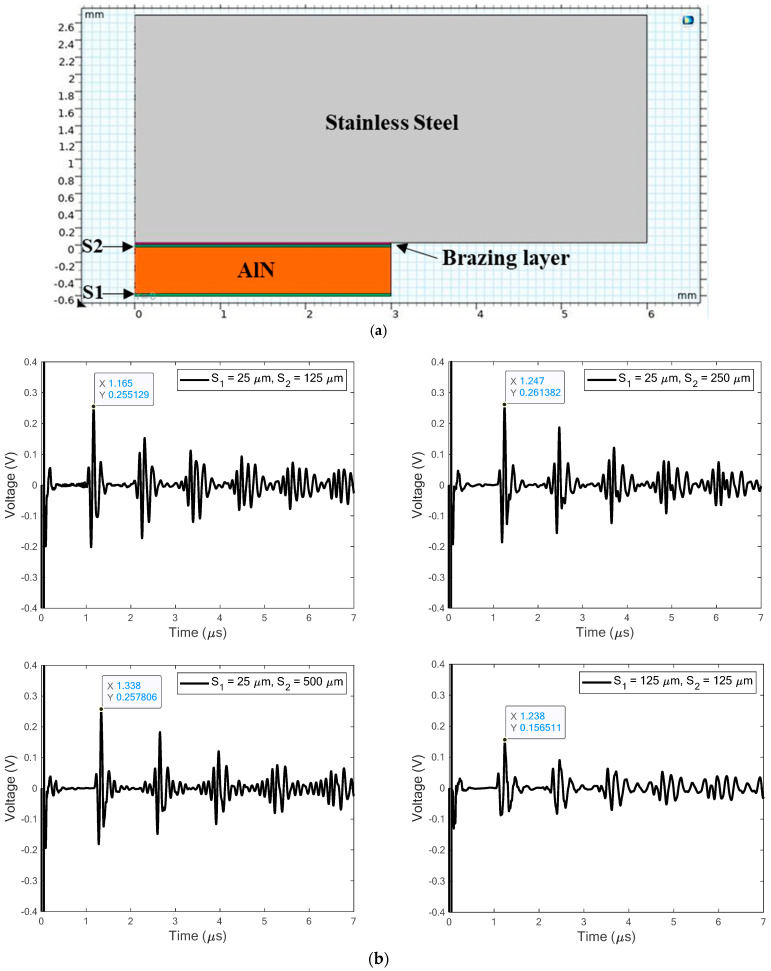
Electrode thickness effects on received signals: (**a**) FEA model with electrodes S_1_ and S_2_, (**b**) A-scans for different thicknesses of electrodes S_1_ and S_2_.

The Ni electrodes are 25 μm thick in the prior simulations. The second question for the model to resolve is whether electrode thickness affects the received signal. This question arises because preferred transducer processing methods may result in different electrode thicknesses or be a poorly controlled process. Defining the free (positive) electrode as S1 and the confined (negative) electrode as S2 in Figure 5a, the electrode thickness is somewhere between 25 and 500 μm, as specified by the four combinations in Figure 5b. Of course, when the S2 electrode is thicker, the TOA is later because the path is longer. Added electrode mass reduces the resonant frequencies of the transducer [14], as evident from the frequency content of a received wave packet (although not shown here). Increasing the thickness of the free electrode S1 from 25 to 125 μm gives an observable change to the wave packet. However, changing the thickness of electrode S2 from 25 to 500 μm did not, although the TOA is larger.

#### 2.2.4. Effect of Coupling Material

**Figure 6:**
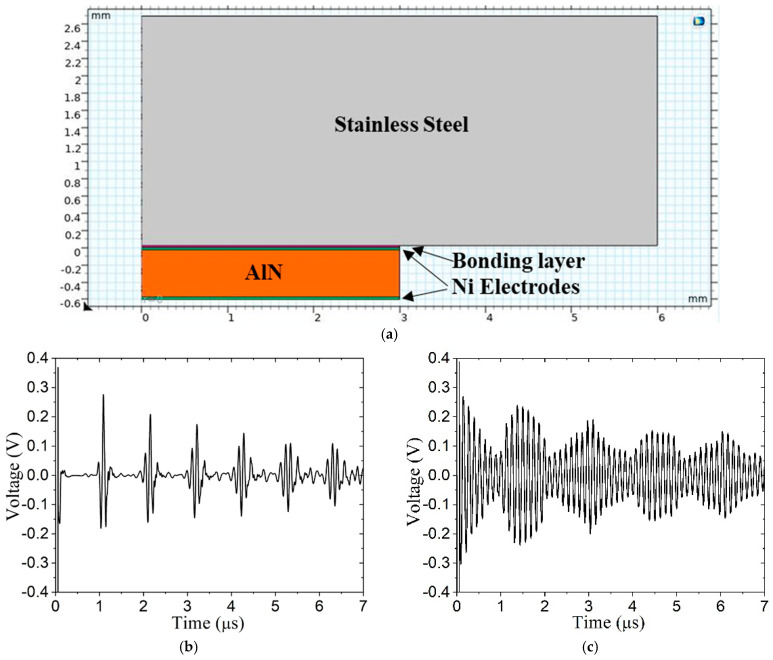
Bonding layer effect: (**a**) model with 10 μm electrodes and 10 μm bonding layer, (**b**) braze bond layer, and (**c**) salol bond layer. Properties of braze are given in Table 3.

Harsh environments significantly limit the materials that can be used to couple the transducer to the vessel wall. Prior simulations have used a braze for that reason. However, preliminary laboratory testing under less harsh conditions may consider adhesives such as epoxy or cyanoacrylate (super glue) or phenyl salicylate (salol). Related studies [15] have been conducted for guided wave transducers for structural health monitoring. Here, the braze material is replaced with the low-melting-point salol material to assess preliminary laboratory testing. The material properties representative of salol are ρ = 1400 kg/m^3^, *E* = 3.7 GPa, and ν = 0.39. The salol thickness is 10 μm, as is the electrode thickness. Figure 6 shows the 2D model and comparable A-scans for braze and salol coupling. Clearly, the salol does not enable the transducer to function properly. Instead, it exhibits significant ringing that obscures the wave propagation in the wall. The signal suggests that the echoes recur in 1.5 μs intervals, in contrast to the 0.93 μs intervals associated with the wave speed in the wall (*c*_L_ = 5718 m/s).

#### 2.2.5. Effect of Grain Coarsening

**Figure 7:**
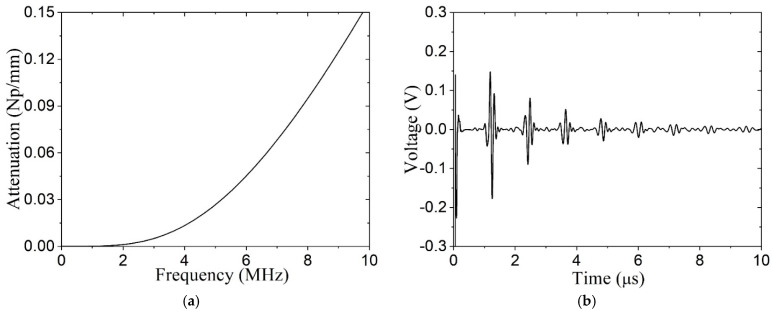
Grain coarsening effect in vessel wall: (**a**) attenuation in stainless steel based on 127 μm mean grain size, (**b**) A-scan signal predicted with attenuation.

Brazing the transducer to the vessel wall entails heating the wall. Microscopy revealed that the average grain size in the stainless-steel wall increased from ~20 to 127 μm due to the thermal aging (temperature increased up to ~316 °C). The large grains attenuate high-frequency ultrasonic waves due to internal scattering. Based on measured attenuation, characterized by Weaver’s model [16], we added a damping coefficient to the simulation (Figure 7a). The received A-scan for the model including attenuation is provided in Figure 7b, which shows that the reverberations ring down faster than previously.

### 2.3. Laboratory Testbed

**Figure 8:**
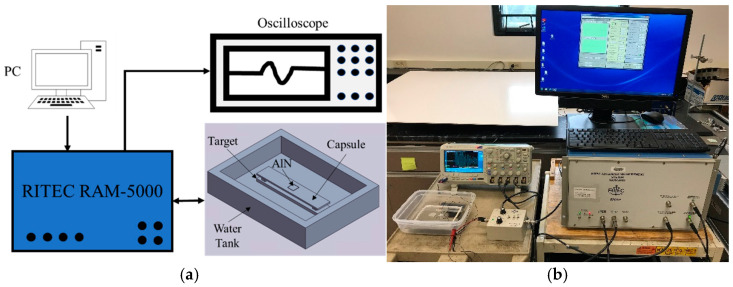
Pulse-echo immersion test laboratory setup: (**a**) schematic, (**b**) photograph.

Our laboratory setup for pulse-echo ultrasonic testing is shown in Figure 8. The primary components are as follows: a personal computer to control the test, the high-power RAM-5000 pulser/receiver (Ritec Corp., Warwick, RI, USA), an oscilloscope to view and record the received signals, and a water tank for immersion testing. Within the filled water tank are the following:

- A surrogate for the vessel wall (henceforth known as the capsule), whose top surface is dry and has an AlN chip coupled to it with conductive silver paste. The chip dimensions are 6 × 6 × 0.55 mm, and the stainless-steel capsule thickness is 2.66 mm.
- A metal target that rests on the bottom of the water tank.
- Two blocks that set the water path between the capsule and target at one of three dimensions—2.33, 2.96, or 6.68 mm.
- Tests were conducted without and with an anomaly on the target’s surface. When the anomaly is present, it is positioned directly in line with the transducer.

Although not shown in Figure 8, as stated earlier, some tests were conducted using the OmniScan pulser/receiver (Olympus Corp., Center Valley, PA, USA) instead of the RAM-5000. The OmniScan sends the negative square wave noted in Section 2.2.1. The RAM-5000, however, sends a toneburst signal. We used a 1.5 cycle toneburst, having a central frequency of 10 MHz after a 2 μs time delay. Furthermore, 60 dB gain was applied to the received signal by the RAM-5000 receiver.

The AlN chip has a much higher electrical Impedance than the 50-Ω transducers that the RAM-5000 is designed to drive. Thus, an impedance matching network can be used. In addition, a diplexer is used to protect the receiver and oscilloscope from the high drive voltage sent to the AlN chip. A high-voltage signal can be sent to the AlN chip because it has no Curie temperature. Finally, harsh environments place demands on cabling that were not addressed in these preliminary laboratory tests.

## 3. Results

The results for pulse-echo ultrasonic immersion testing are described from finite element analysis as well as the laboratory setup.

### 3.1. Immersion Test Model

**Figure 9:**
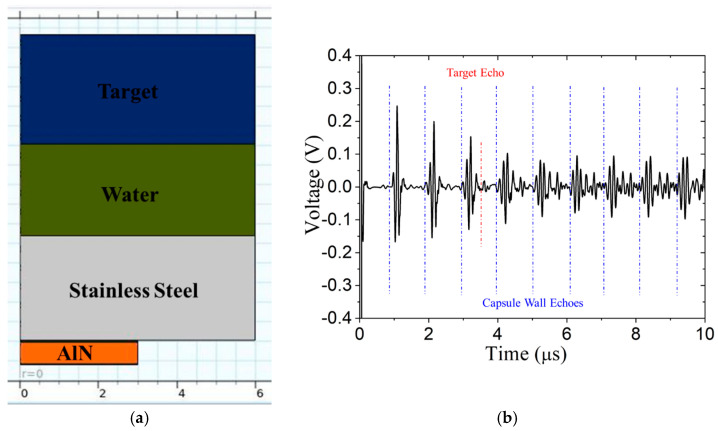
Pulse-echo signal given a 2.1 mm water path: (**a**) model, (**b**) A-scan dominated by vessel wall reverberation. Predicted arrival times of vessel wall reverberations and target echo are shown in blue and red, respectively.

The laboratory setup shown in Figure 8 is modelled by adding water and a 2.8 mm thick zircaloy-4 target to the previously described finite element model. The model shown in Figure 9a has a 2.1 mm water path between the stainless steel capsule and the target. There is no backing layer on the AlN transducer, and grain coarsening associated with brazing is not considered. The received signal at room temperature is shown in Figure 9b. Reverberations having a fixed interval are apparent and highlighted by dashed blue vertical lines. Presuming the longitudinal wave speed in the capsule is 5718 m/s, the interval is 0.93 μs. The second, third, and fourth echoes have decreasing amplitudes, and then the echo amplitude becomes relatively constant. The coherent noise between echoes increases with time, even though there is no random noise injected into these simulations. The echo from the target is indistinguishable from the coherent noise between echoes. The calculated TOA of the target echo is shown as a dashed red vertical line (at 3.75 μs).

**Figure 10:**
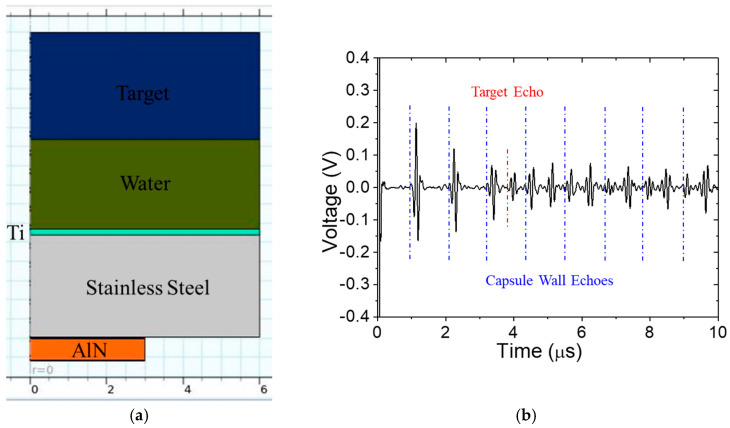

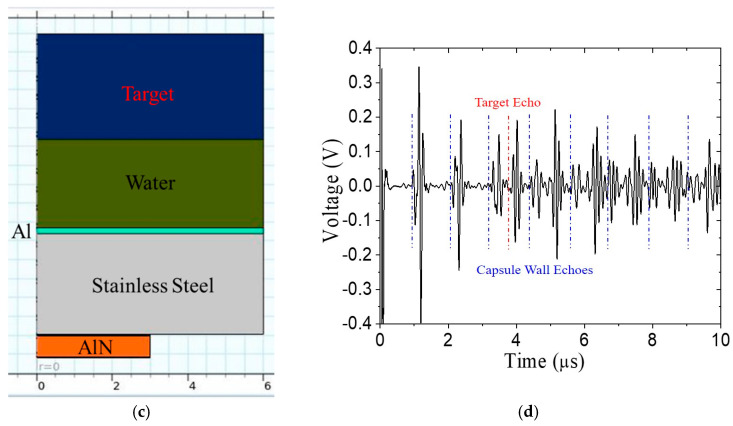
Pulse-echo signal at room temperature with a 2.1 mm water path: Ti matching layer (**a**) model, (**b**) signal and Al matching layer (**c**) model, (**d**) signal. Predicted arrival times of vessel wall reverberations and target echo are shown in blue and red, respectively.

**Figure 11:**
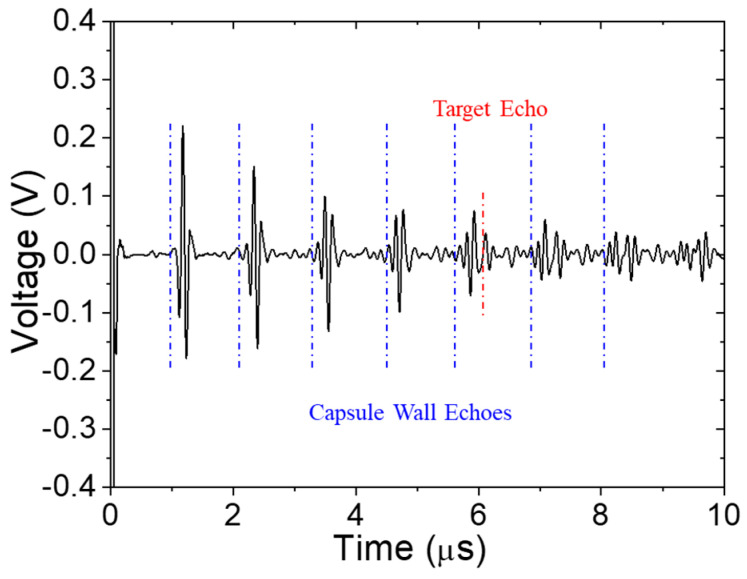
Pulse-echo signal at elevated temperature (316 °C) with a 2.1 mm water path and Ti matching layer. Predicted arrival times of vessel wall reverberations and target echo are shown in blue and red, respectively.

The large acoustic impedance, *Z*, mismatch between stainless steel and water results in reverberations in the capsule (this mismatch is, however, less than that between stainless steel and air in Section 2.2). An impedance matching layer could reduce the reverberation. The acoustic impedance of the matching layer should be $Z_{match}=\sqrt{Z_{capsule}Z_{water}}$ according to Rathod [17]. Acoustic impedance values at room temperature are given in Table 4. Thus, a quarter-wavelength matching layer is joined to the stainless steel. Titanium and aluminum matching layers are investigated at room temperature (20 °C), with the results shown in Figure 10. Compared to Figure 9b, the amplitude of the target echo in Figure 10b is roughly two-times higher, but it is still obscured by the reverberation. If the same Ti matching layer is used at 316 °C, the reverberation interval increases somewhat, and the target echo TOA is considerably later because the wave speed in water decreases much more than it does in stainless steel. The received A-scan is shown in Figure 11. For the same water path of 2.1 mm, the target echo now coincides more closely with a reverberation instead of occurring between them. This is obviously not optimal, and either the water path or the wall thickness should be adjusted, if possible.

**Figure 12:**
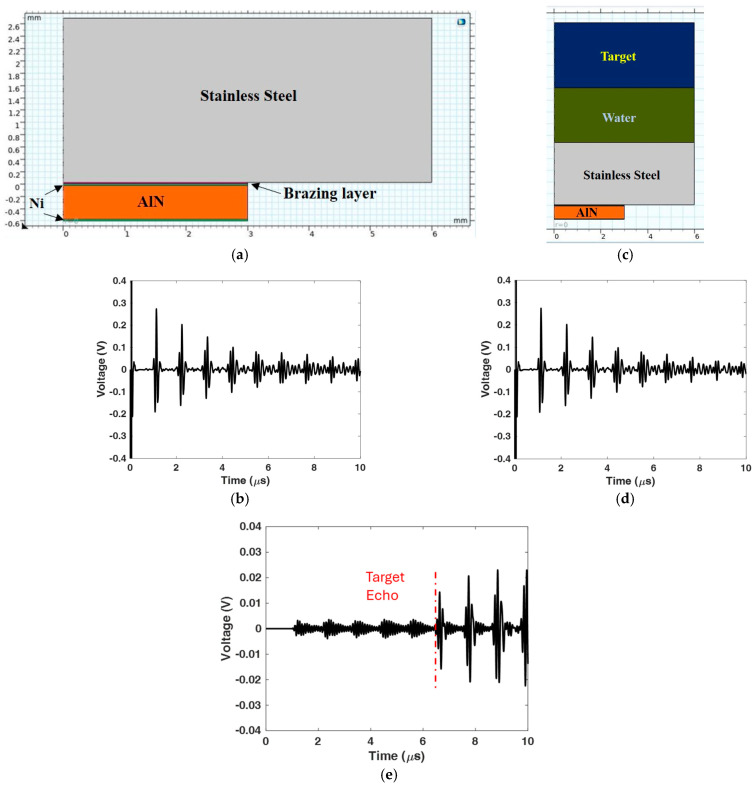
Signal subtraction method removes reverberations: (**a**) model without target; (**b**) baseline signal PSE_1_; (**c**) model with target; (**d**) signal PSE_2_; (**e**) difference signal DS = PSE_2_ – PSE_1_. Notice the scale of the difference signal DS is an order of magnitude smaller than PSE_1_ and PSE_2_.

In all the predicted pulse-echo signals, the target echo, which we seek to detect clearly, is obscured by the reverberation in the capsule wall. Presuming stable operating conditions, it should be possible to subtract the reverberations out of the received A-scan. Thus, we will start by acquiring a baseline pulse-echo signal from the test system without a target present, called PES_1_. After inserting the target, another pulse-echo signal PES_2_ is acquired. The difference signal, DS = PES_2_ – PES_1_, should effectively be free of the unwanted reverberations. A sample difference signal is illustrated in Figure 12. In the absence of identical reverberations in signals PES_1_ and PES_2_, the reverberations will not entirely cancel, although they are significantly reduced, as shown in Figure 12e, prior to 6 μs. Notice the scale of the difference signal DS is an order of magnitude smaller than for PSE_1_ and PSE_2_. The target echo arrives after 6 μs, followed by its reverberation in the vessel wall. Although the reverberations are effectively cancelled out, even in a noise-free finite element simulation, they are not identically cancelled. In some instances, it may be beneficial to scale the baseline signal PES_1_ to eliminate the capsule wall echo closest to the predicted TOA of the target echo. If the phases of signals PES_1_ and PES_2_ are misaligned, then stretching or contracting the baseline may be beneficial. Phase differences in PES_1_ and PES_2_ can, in principle, be minimized by acquiring them under constant conditions. However, this is often impractical in structural health monitoring applications. Thus, temperature compensation strategies have been developed, as described by Cawley [18] and the literature cited therein. An alternative solution is to use an infinite impulse response filter of the form,

$$y\left(n\right)=w\left(n\right)+\alpha w(n-\Delta )$$

where $w\left(n\right)$ and $y\left(n\right)$ are the input and output signals, respectively, α is an attenuation coefficient, and Δ is the time difference between reverberations. The parameters α and Δ can be adjusted to account for changes in environmental conditions that would affect the wave propagation. However, we do not investigate this filter here.

The target’s surface may be susceptible to anomalies, such as corrosion pits, scale formation, biological fouling, and swelling. In this study, we use an artificial reflector as a well-controlled surrogate for a surface anomaly. The reflector is a metal disc joined to the surface of the target. The disc is aligned with the center of the transducer. We start by analyzing a disc that is 0.635 mm thick, 4 mm in diameter, and the same material as the target.

**Figure 13:**
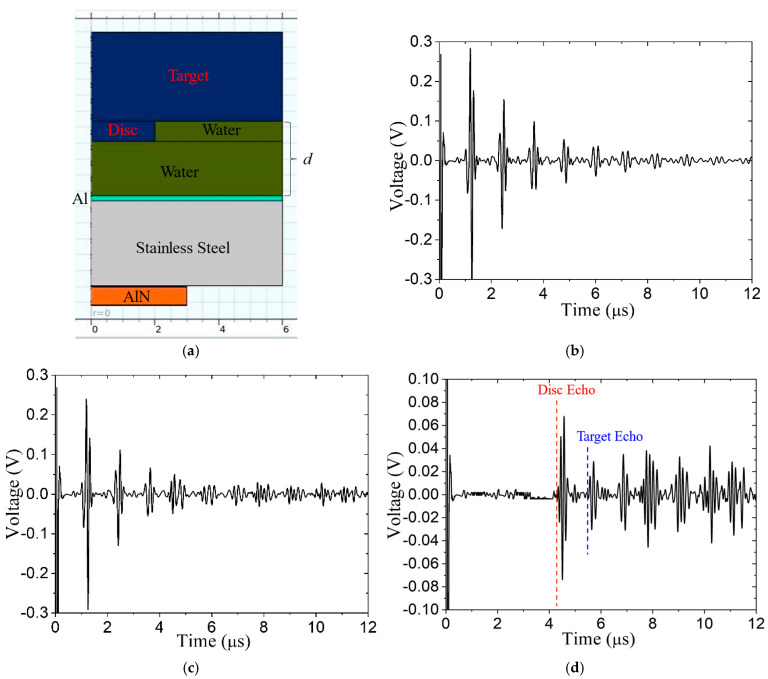
Signal subtraction method removes reverberations: (**a**) model with 0.635 mm thick disc target surface anomaly, (**b**) signal PES_1_, (**c**) signal PES_2_, and (**d**) difference signal DS. Predicted arrival times of target echo and disc echo are shown in blue and red, respectively.

Figure 13a shows the axisymmetric model with a 2.33 mm water path that is impinged upon by an Al matching layer on one side and the disc on the other. Figure 13b,c show the signals PES_1_ (baseline) and PES_2_, respectively, and Figure 13d shows the difference signal DS. In this case, the baseline subtraction very well eliminates the reverberations, and both the echoes from the disc surface and the target surface are readily identifiable.

**Figure 14:**
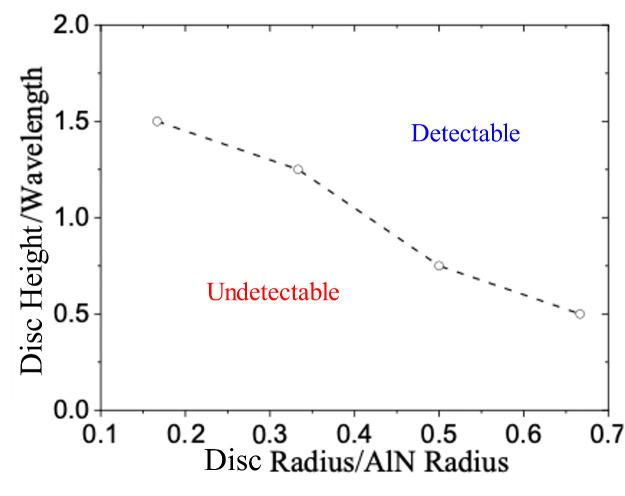
Effect of disc-shaped anomaly radius and height on echo detectability.

Another important measure of the NDT system is the detection sensitivity, namely its ability to detect the smallest surface anomaly. Previous examples consider one disc size, while the disc radius and height both affect its detectability. Thus, a series of COMSOL models were built with random disc radius and height. After post-processing all cases, a boundary line between the detectable disc size region and the undetectable disc size region was determined, as shown in Figure 14. In Figure 14, the *x*-axis is the disc radius normalized by the AlN wafer radius, and the *y*-axis is the disc height normalized by the wavelength in the water (specifically, 0.2 mm). If the disc geometry plots in the upper-right region, it will be detectable using the signal subtraction technique; otherwise, it cannot be detected. The detectability threshold is based on the signal-to-noise ratio being greater than two in the finite element analysis, where there is no random noise. Thus, this is the best-case scenario.

### 3.2. Laboratory Testing

**Figure 15:**
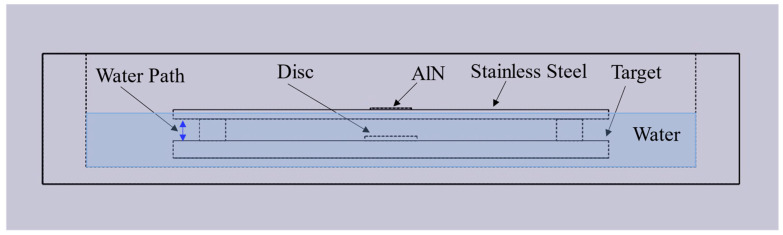
Laboratory setup for water paths of 2.33, 2.96, and 6.68 mm.

The laboratory setup is shown in Figure 15. The AlN transducer is coupled to silver paste, there is no backing or matching layer, and three different water paths are used based on different block sizes. The whole test assembly is immersed in water, except for the top of the stainless steel capsule, where the AlN transducer is mounted. The AlN transducer is well aligned with the disc surface anomaly on the target before the experiment. The target is removed when collecting the reference signal for signal subtraction.

**Figure 16:**
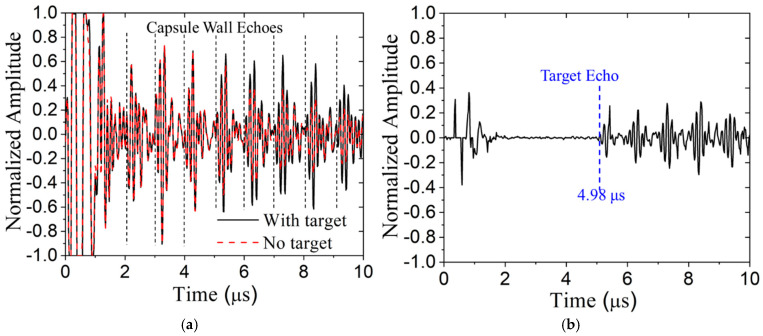
Signal subtraction method with signals from OmniScan: (**a**) 2.96 mm water path, (**b**) difference signal, DS. Predicted target time of arrival shown in blue.

To experimentally demonstrate the effectiveness of the proposed signal subtraction technique, the Olympus OmniScan pulser/receiver was used to acquire the baseline signal without the target (PES_1_) and with it (PES_2_). The normalized signals are shown in Figure 16a, and the difference signal, DS, is shown in Figure 16b. The target echo is not evident in Figure 16a (its TOA is 4.98 μs); however, it is clearly visible in Figure 16b, as are reverberations of the target echo in the capsule wall. Apparently, there is randomness in the early signal, prior to 2 μs that is not eliminated by subtraction. This is not surprising because that early signal is dominated by the breakthrough and transducer ringing.

**Figure 17:**
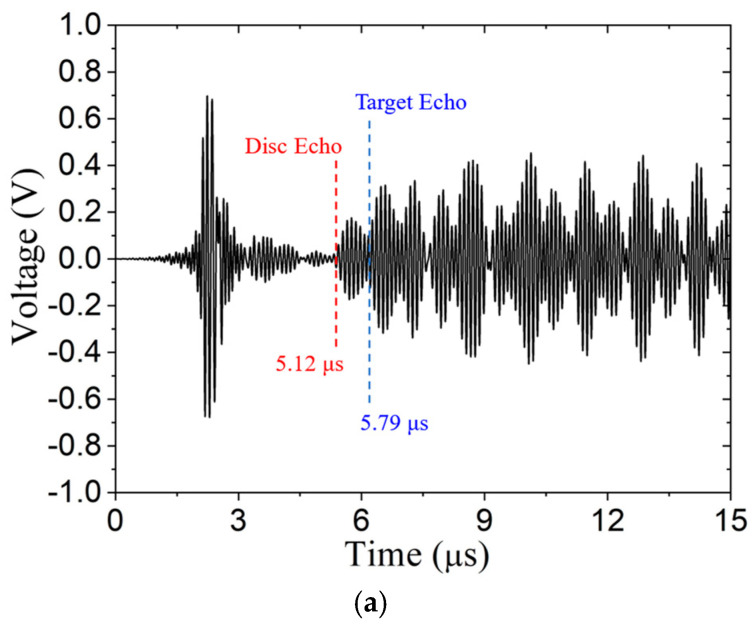

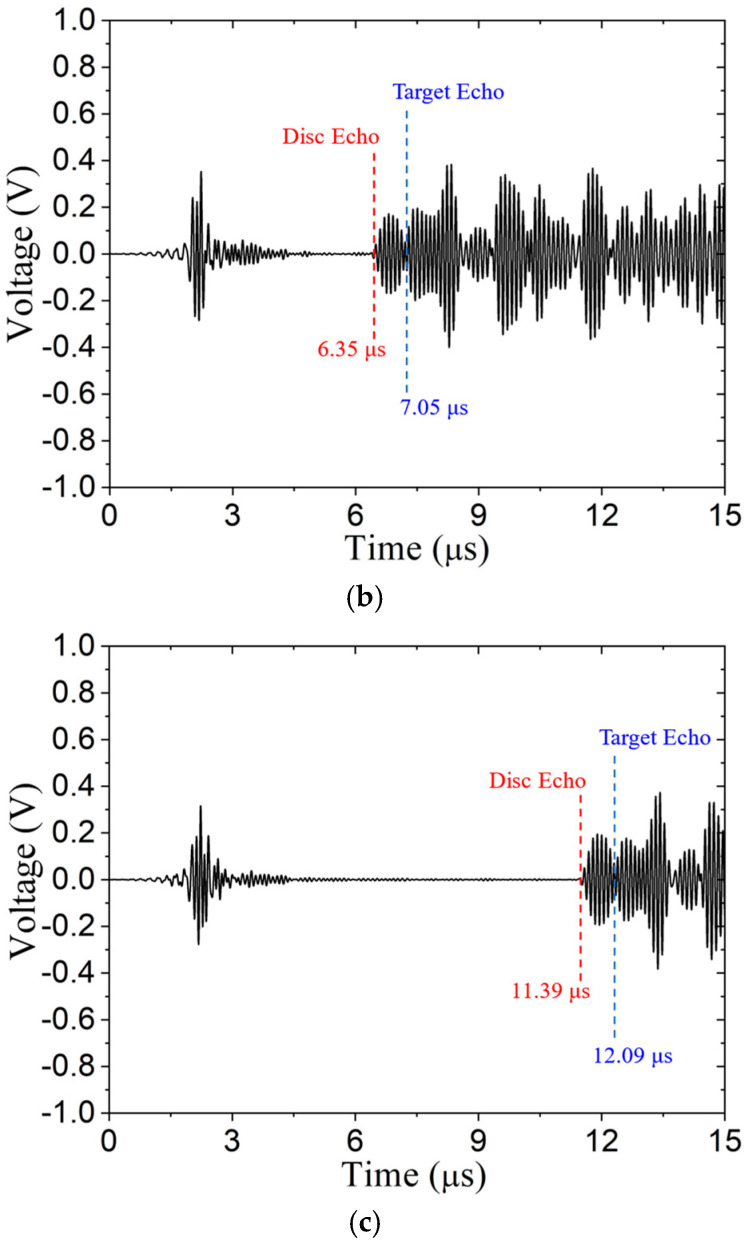
Signal subtraction method with signals from RAM-5000 using a 2.0 μs delay and water paths of: (**a**) 2.33 mm, (**b**) 2.96 mm, and (**c**) 6.68 mm. Predicted time of arrival of target echo and disc anomaly echo shown in blue and red, respectively.

To further illustrate the capability of the ultrasonic pulse-echo system to detect a 0.635 mm thick disc on the target’s surface, the RAM-5000 instrument is used to apply high voltage to the AlN transducer. The driving signal is a 1.5-cycle toneburst, having a 10 MHz central frequency with a 2 μs delay, and the received signal is amplified 60 dB. Immersion testing is performed with water paths of 2.33, 2.96 and 6.68 mm. The difference signals (DSs) are shown in Figure 17. TOA computed based on assumed wave speeds are listed in Table 5, and the TOA for the disc echo and the target echo are identified in Figure 17. Based on these results, we concluded that the metal target and the disc on the target’s surface are detected with the pulse-echo system. From the A-scans in Figure 17, we can quantify the signal-to-noise ratio (SNR) of the target echo and compare it with the results from the OmniScan instrument (see Table 6). Figure 17 indicates that SNR improves with water path length.

## 4. Discussion

We considered situations where data from the reflections of an immersed target in a harsh environment provide useful information about system operations or the health status by nondestructive evaluation. These measurements can provide unique and critical information in harsh environments. Specifically, the ultrasonic wave path includes a vessel wall and a fluid. That is, a piezoelectric wafer is bonded to the exterior of the vessel and used in pulse-echo mode. As material selection for harsh environments has been covered elsewhere; several other aspects that influence the data quality were investigated. The primary underlying issue is that the acoustic impedance mismatch between the vessel wall and the fluid causes significant reverberation in the vessel work that obscures the echo from the target. The investigation used finite element analysis to show the following:

- How effectively a viscous backing material on the piezoelectric wafer decreases transducer ringing;
- That the thickness of the free electrode on the piezoelectric wafer can eventually become large enough to change the dynamic response of the transducer, while the thickness of the sandwiched electrode mostly affects the time of arrival;
- A solid couplant can greatly influence the received signal—if it is too compliant, then ringing occurs, and if it employs thermal processes (such as brazing), then it can change the grain size of the vessel wall and affect attenuation;
- That the target echo can be completely obscured by vessel wall reverberations, but an acoustic matching layer reduces reverberations enough that the target echo becomes visible, albeit the signal-to-noise ratio is low;
- A very simple subtractive signal processing approach can sufficiently reduce the vessel wall reverberations from the signal to provide a very reasonable signal-to-noise ratio for the target echo;
- When using the subtractive signal processing, a disc-like surface anomaly could be detected along with the target echo.

Experiments were conducted to demonstrate that the finite element results translate into the laboratory. The subtractive signal processing was effective, and a disc-like surface anomaly was detected.

## Figures and Tables

**Table 1:** COMSOL interfaces used in the model.

Module	Component	Material
Solid Mechanics	vessel wall, bonding layer, electrode, target	stainless steel, various, various, zircaloy
Piezoelectricity	transducer	aluminum nitride
Acoustics	fluid	water
Electrical	driving circuit	n.a.

**Table 2:** Geometries of components in the 3D and 2D models.

Component	3D Model	2D Model
Transducer	6 × 6 × 0.55 mm	Ø6 × 0.55 mm
Electrode	6 × 6 × 0.025 mm	Ø6 × 0.025 mm
Braze	6 × 6 × 0.025 mm	Ø6 × 0.025 mm
Vessel Wall	8 × 8 × 2.66 mm	Ø12 × 2.66 mm

**Table 3:** Material properties.

Component	Material Properties at 20 °C			Material Properties at 316 °C		
Transducer (AlN)	Ref [8]			Ref [8]		
Fluid (water)	ρ = 1000 kg/m^3^, *c*_L_ = 1490 m/s			ρ = 750 kg/m^3^, *c*_L_ = 1020 m/s		
Backing (Si-W)	ρ = 2500 kg/m^3^, *E* = 3.6 Gpa, α=46 dB/mm			n.a.		
	ρ (kg/m^3^)	*E* (Gpa)	ν (-)	ρ (kg/m^3^)	*E* (Gpa)	ν (-)
Electrode (Ni)	8890	205	0.31	8700	189	0.31
Braze (Bni-6)	8200	160	0.31	8200	160	0.31
Wall (stainless steel)	8027	195	0.30	7889	172.4	0.30

**Table 4:** Acoustic impedance *Z* (MRayl).

Stainless Steel	Water	Match	Titanium	Aluminum
42.79	1.5	8.01	25.08	15.23

**Table 5:** Experimental time of arrival (TOA) compared to calculated values.

Case	Water Path (mm)	Disc Echo TOA (μs)		Target Echo TOA (μs)	
		Theory	Experiment	Theory	Experiment
1	2.33	3.08	3.12	3.95	3.79
2	2.96	4.30	4.35	5.00	5.05
3	6.68	9.21	9.39	9.90	10.09

**Table 6:** Signal-to-noise ratio of target echo.

OmniScan	RAM-5000
9	20

## Data Availability

The original contributions presented in the study are included in the article; further inquiries can be directed to the corresponding author.
